# Nitrous Oxide Production in a Granule-based Partial Nitritation Reactor: A Model-based Evaluation

**DOI:** 10.1038/srep45609

**Published:** 2017-04-03

**Authors:** Lai Peng, Jing Sun, Yiwen Liu, Xiaohu Dai, Bing-Jie Ni

**Affiliations:** 1 State Key Laboratory of Pollution Control and Resources Reuse, College of Environmental Science and Engineering, Tongji University, Shanghai 200092, P.R. China; 2 Research group of Sustainable Energy, Air and Water Technology, Department of Bioscience Engineering, University of Antwerp, Antwerp 2020, Belgium; 3 Centre for Technology in Water and Wastewater, School of Civil and Environmental Engineering, University of Technology Sydney, Sydney, NSW 2007, Australia

## Abstract

Sustainable wastewater treatment has been attracting increasing attentions over the past decades. However, the production of nitrous oxide (N_2_O), a potent GHG, from the energy-efficient granule-based autotrophic nitrogen removal is largely unknown. This study applied a previously established N_2_O model, which incorporated two N_2_O production pathways by ammonia-oxidizing bacteria (AOB) (AOB denitrification and the hydroxylamine (NH_2_OH) oxidation). The two-pathway model was used to describe N_2_O production from a granule-based partial nitritation (PN) reactor and provide insights into the N_2_O distribution inside granules. The model was evaluated by comparing simulation results with N_2_O monitoring profiles as well as isotopic measurement data from the PN reactor. The model demonstrated its good predictive ability against N_2_O dynamics and provided useful information about the shift of N_2_O production pathways inside granules for the first time. The simulation results indicated that the increase of oxygen concentration and granule size would significantly enhance N_2_O production. The results further revealed a linear relationship between N_2_O production and ammonia oxidation rate (AOR) (R^2^ = 0.99) under the conditions of varying oxygen levels and granule diameters, suggesting that bulk oxygen and granule size may exert an indirect effect on N_2_O production by causing a change in AOR.

Nitrous oxide (N_2_O) is of significant environmental concern since it is not only a potent greenhouse gas (approximately 265-fold stronger global warming potential than carbon dioxide), but also responsible for stratospheric ozone depletion^1^,^2^. N_2_O can be produced during wastewater treatment, especially from biological nitrogen removal (BNR). Ammonia oxidizing bacteria (AOB) have been considered as the main contributor to N_2_O production via two pathways: (i) the sequential reductions from nitrite (NO_2_^−^) to nitric oxide (NO) and to N_2_O as the end product, termed as AOB denitrification^3^,^4^,^5^; (ii) N_2_O as a side product during incomplete oxidation of hydroxylamine (NH_2_OH) to NO_2_^−^ 6,7. N_2_O production by AOB was demonstrated to be related to dissolved oxygen (DO), nitrite, pH, ammonia loading, alkalinity, etc^3^,^4^,^8^,^9^,^10^,^11^,^12^. The site preference (SP), a promising isotopic technique for source identification of N_2_O production, was calculated as the difference between alpha and beta isotopomer signatures. SP has a major advantage of being independent of the precursors^13^. DO and nitrite played important roles in shifting the contribution of AOB denitrification pathway and NH_2_OH oxidation pathway^13^,^14^,^15^.

During past years, an innovative technology based on shortcut nitrification (partial nitritation, PN) and anaerobic ammonium oxidation (anammox) offers a superior alternative to conventional processes in WWTPs (i.e. nitrification-denitrification), saving 30–40% of the overall costs due to a significantly lower oxygen (O_2_) demand, minimized external carbon requirements and sludge yield^16^. Granule-based reactors have been widely used for PN processes owing to high specific nitrification rate, good settling velocity and efficient biomass retention^17^. However, N_2_O emissions in the partial nitritation/anammox processes have been demonstrated to be significant, particularly in the partial nitritation process^18^,^19^,^20^. The results from SP analysis in the study of Rathnayake *et al*.^17^ revealed that AOB denitrification pathway and NH_2_OH oxidation pathway made comparable contributions to N_2_O production from a lab-scale granule-based PN reactor, which was fed with synthetic wastewater without organic carbon supplement. Higher N_2_O production in autotrophic PN granules was observed at higher bulk DO concentration and pH of 7.5, which was mostly contributed by *Nitrosomonas europaea* in oxic layer^21^. Pijuan *et al*.^22^ observed a much higher N_2_O emission in sequencing batch mode compared to continuous airlift mode and a increase of N_2_O upon decreasing DO levels in granular bioreactors. Nevertheless, more research needs to be conducted to elucidate the involved mechanisms in such a system, including the source identification of N_2_O production and its correlation to multiple parameters.

Mathematical modeling serves as a powerful tool for design and operation of wastewater treatment systems and provides insights into the biochemical pathways. Mathematical models describing N_2_O production by AOB from single pathways or two pathways have been developed and assessed under varying operational conditions (i.e. DO, nitrite, inorganic carbon, etc.)^23^,^24^,^25^,^26^,^27^. Peng *et al*.^28^ further compared these single-pathway models and two-pathway model to identify the applicable region, defined by varying DO and nitrite levels, for each of the models. Besides the suspended-growth systems, mathematical model was also used to predict N_2_O production from biofilm systems. Peng *et al*.^29^ applied a single-pathway model to investigate N_2_O production from biofilm reactor performing partial nitritation and anammox (PNA) and found the optimal conditions at low ammonium concentration (<50 mg N/L), moderate DO level (at around 0.5 mg O_2_/L) and biofilm thickness of 1000 μm. Sabba *et al*.^30^ revealed that the diffusion of NH_2_OH to anoxic region of biofilm significantly enhanced N_2_O emission using a two-pathway N_2_O model. These previous work clearly demonstrated the importance of mathematical modeling for improving the understanding of N_2_O production mechanisms^26^,^27^. However, mathematical modeling of N_2_O production in granule-based PN bioreactor using the two-pathway N_2_O model is currently unavailable. Van Hulle *et al*.^31^ performed numerical simulation of N_2_O/NO emissions from single-stage granular PNA. However, only one single N_2_O pathway was applied and no verification was performed with experimental data. Pijuan *et al*.^22^ correlated N_2_O production in a continuous granular airlift nitritation reactor to DO levels. And these experimental data was further compared to predictions of a granular N_2_O model under varying DO concentrations^30^. However, the involved mechanism including the substrate conversion (i.e. NH_2_OH, electron, etc.) inside granule and the impact of key parameters on N_2_O production as well as the two pathways are unclear. Therefore, it is of great interest to investigate the N_2_O production and the shift of pathways in granule-based PN reactor as well as inside aerobic granules using the two-pathway N_2_O model.

In this work, the previously-established two-pathway N_2_O model incorporating both AOB denitrification and NH_2_OH oxidation pathways^26^ was applied to describe the N_2_O production in granule-based PN reactor. The validity and applicability of the N_2_O model were tested by comparing simulations with process data from a lab-scale granular PN sequencing batch reactor (SBR) and isotopic data on pathway contributions. The dependency of N_2_O production on oxygen concentration and granule size as well as the mechanisms involved were evaluated.

## Results and Discussion

### Model evaluation with experimental data

**Figure 1:**
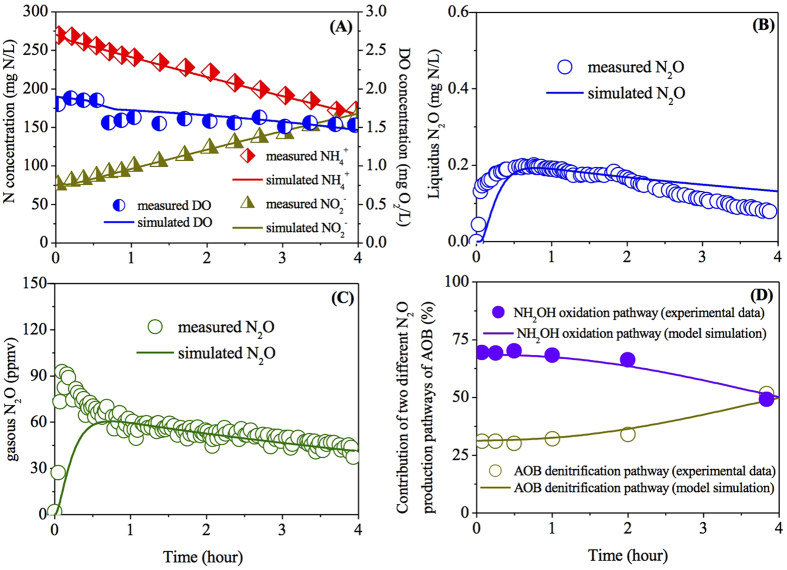
Model calibration using experimental data from a partial nitritation granular reactor: (**A**) NH_4_^+^ and NO_2_^−^; (**B**) dissolved N_2_O; (**C**) N_2_O emission; and (**D**) contribution by two N_2_O pathways.

Model evaluation of this work involved adjusting key parameter values for the nitrogen conversion (*r*_*NH*3,*ox*_ and *K*_*O*2,*NH*3_) and N_2_O production processes (*r*_*NO*2,*red*_, *r*_*NO,red*_, *K*_*I*,1_ and *K*_*I*,2_). The model prediction and experimentally measured data during a typical experimental SBR cycle are shown in Fig. 1, including the nitrogen, DO (Fig. 1A) and dissolved N_2_O in bulk liquid (Fig. 1B), N_2_O emission in the off-gas (Fig. 1C) and the relative contributions of the AOB denitrification pathway and the NH_2_OH oxidation pathway (Fig. 1D). The estimated values of *r*_*NH*3,*ox*_, *r*_*NO*2,*red*_, *r*_*NO,red*_ and *K*_*O*2,*NH*3_ are 0.1, 0.077, 0.0059 mg-N/(mg-COD*h) and 5 mg O_2_/L, respectively. These values are comparable with those reported in the literature, which are 0.15^26^, 0.039^13^, 0.00012^13^ mg-N/(mg-COD*h) and 2.1^11^ mg O_2_/L. Figure S1 (in the Supplementary Material) shows the two joint 95% confidence regions for parameter combinations, together with the confidence intervals for all the parameters. Overall, the 95% confidence regions for the two pairs are small, with mean values lying at the center. The 95% confidence intervals for all the single parameters are also small (generally within 10% of the estimated values). These indicate a high-level identifiability and reliability of the estimated values.

Most of oxidized NH_4_^+^ was converted to NO_2_^−^ (Fig. 1A) with minimum conversion to NO_3_^−^ (data not shown). The DO profile displayed a decreasing trend within the range of ~1.5–~1.9 mg O_2_/L. The model simulation results with the calibrated parameters (*r*_*NH*3*,ox*_ and *K*_*O*2*,NH*3_) applied were in good agreement with the measured N and DO dynamics (Fig. 1A). Both dissolved N_2_O (Fig. 1B) and emitted N_2_O gas (Fig. 1C) increased initially and gradually decreased after reaching peak. The N_2_O emission process was modeled through the N_2_O volumetric transfer coefficient and liquid-phase N_2_O produced by the two pathways. The developed model could generally describe the decreasing trends of both N_2_O profiles. However, the N_2_O peak in gas phase (Fig. 1C) was slightly underestimated by the model, possibly due to the potential gas measurement error considering its inconsistence with the observed liquid phase N_2_O profile, which could not be accurately captured by the model.

Based on analysis of SP measurement, the contributions of each N_2_O pathway were shown in Fig. 1D, where the contribution of NH_2_OH oxidation pathway was initially predominant, but decreased over time and finally was comparable with that of AOB denitrification pathway at the end of cycle. The calibrated N_2_O model properly captured these shifts of N_2_O pathways over time. Based on the predictions by the same N_2_O model, it was seen that AOB denitrification pathway was dominant over NH_2_OH oxidation pathway when nitrite varied between 0 and 700 mg N/L in suspend-growth PN reactor^26^. The inconsistent observations may result from the different operational conditions. For instance, the DO levels in this study (1.5–1.9 mg O_2_/L) are substantially higher than those in Ni *et al*.^26^ (~0.55 mg O_2_/L). Higher DO could promote NH_2_OH oxidation pathway, but suppress AOB denitrification pathway^13^. A Haldane-type kinetics (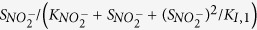) was applied to describe the NO_2_^−^ reduction in both studies. However, the obtained value of nitrite inhibition constant for nitrite reduction was 500 mg N/L in this study, while the value of the same parameter was 48 mg N/L in the study conducted by Ni *et al*.^26^, indicating lowered inhibitory effect of nitrite in this study, possibly due to different microbial community involved.

Apart from the biological N_2_O production pathways, abiotic N_2_O generation from the reaction between NH_2_OH and free nitrous acid (FNA) could be a recognized resource for N_2_O production^32^,^33^. However, compared to these two studies, the NH_2_OH concentration, predicted by the mathematical model, and the FNA, calculated based on pH, temperature and NO_2_^−^ were several orders of magnitude lower. Hence, the contribution of the abiotic N_2_O production is expected to be minimum.

### N_2_O production profile inside granule

**Figure 2:**
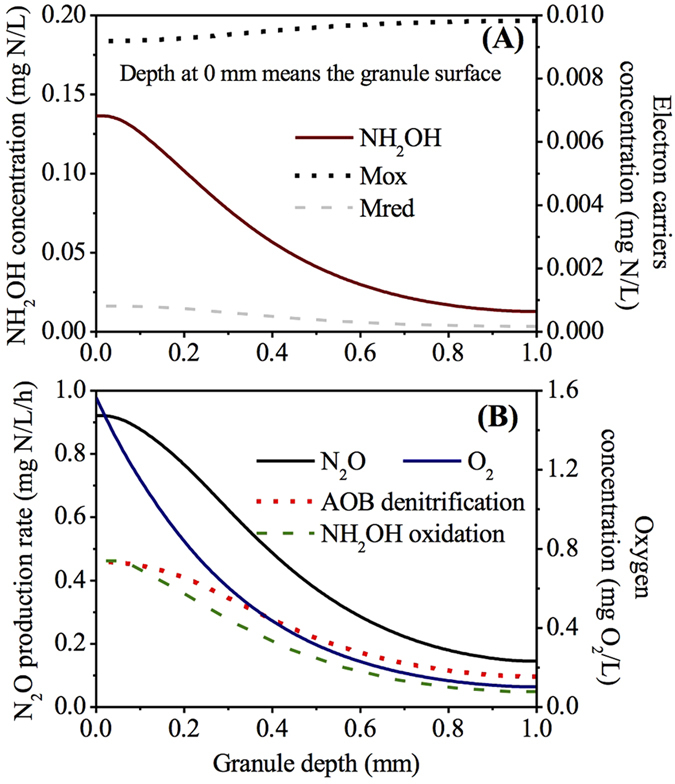
Model simulation results of NH_2_OH and electron carrier distribution (**A**) as well as N_2_O production and oxygen gradient (**B**) inside granule.

In this work, we assume that there is no bacteria growth and AOB is evenly distributed throughout the AOB granule. We admit that this assumption does not necessarily and completely reflect the reality of microbial distribution. We would like to clarify that our model is not meant to describe in full details in AOB granules. Instead, it is meant to be a practically useful tool for predicting N_2_O production as well as production pathway under varying conditions in PN-SBR. It is essential that the model component is kept relatively simple. The assumptions are justifiable since heterotrophic bacteria growing cell lysate is very limited due the lack of organic matter in the influent and AOB are slow growing. The same assumptions have been used in previous study by Sabba *et al*.^30^ for description of N_2_O production from AOB biofilm and AOB granule. The distribution profiles of NH_2_OH, Mox, Mred, O_2_ and N_2_O production rates via the two known pathways within the granule at the end of the cycle are presented in Fig. 2. Point 0 on the x-axis indicates the granule surface, while point 1.0 on the x-axis represents the granule center. Along granule depth from surface to inner part, NH_2_OH decreased rapidly from ~0.13 to ~0.01 mg N/L (Fig. 2A). The Mred concentration gradually decreased from 0 to 1 mm and depleted at granule center, whereas the Mox concentration displayed an opposite trend (Fig. 2A). The O_2_ concentration was ~1.6 mg O_2_/L at the granule surface with lower concentration observed at the inner layer (Fig. 2B).

The distribution of N_2_O production rate matched the predicted substrate stratification (O_2_, NH_2_OH and Mred) of the granule (Fig. 2B). From the outer layer to the inner layer, N_2_O production rates via both pathways diminished (Fig. 2B), possibly due to the fact that the lower oxygen and NH_2_OH levels in the inner layer limited electron supply for both nitrite reduction and NO reduction. Peng *et al*.^13^ observed similar correlation between N_2_O production rate and DO in an enriched nitrifying culture, which was attributed to the increased electron supplying rate due to the fact that a higher DO increased the ammonia and hence NH_2_OH oxidation. However, the model prediction concerning on N_2_O production rate displayed a discrepancy against the observation by Rathnayake *et al*.^17^, which was likely due to the different experimental conditions for the PN-SBR and the flow chamber for microsensor measurement. Sampled granules were positioned with five needles in the flow chamber, where DO and pH were roughly constant. The substrate profiles of DO, N_2_O, NH_4_^+^, NO_2_^−^ and NO_3_^−^ in the PN granules were measured in a synthetic medium containing 3.5 mg N/L NH_4_^+^, 3.5 mg N/L NO_2_^−^ and 0.7 mg N/L NO_3_^−^. In contrast, the SBR was fed with synthetic wastewater containing much higher substrate concentrations (210 mg N/L NH_4_^+^ and 140 mg N/L NO_2_^−^). More importantly, the PN-SBR is a complete mixed compartment with higher microbial activity and more dynamic conditions in terms of pH, DO and nitrogen conversion.

Both AOB denitrification and NH_2_OH oxidation pathways made comparable contributions at the outer granule layer (0–0.1 mm) with more oxygen. However, from 0.1 to 1.0 mm, the AOB denitrification pathway became the main contributor in the presence of limited oxygen. With the aid of mathematical modeling, the N_2_O production via the two known pathways inside PN granule is revealed for the first time in this study. Although there is still a lack of experimental data for validation, the simulation results from granule-based N_2_O model were indeed supported by observations from both pure and enriched cultures, where the decrease of oxygen favored N_2_O production via AOB denitrification pathway over NH_2_OH oxidation pathway^3^,^13^,^34^. Anoxic region with the presence of NH_2_OH in nitrifying biofilm or granule could further lead to substantially higher N_2_O production via AOB denitrification pathway than that in suspended-growth sludge^30^.

### Effects of bulk oxygen and granule size on N_2_O production

**Figure 3:**
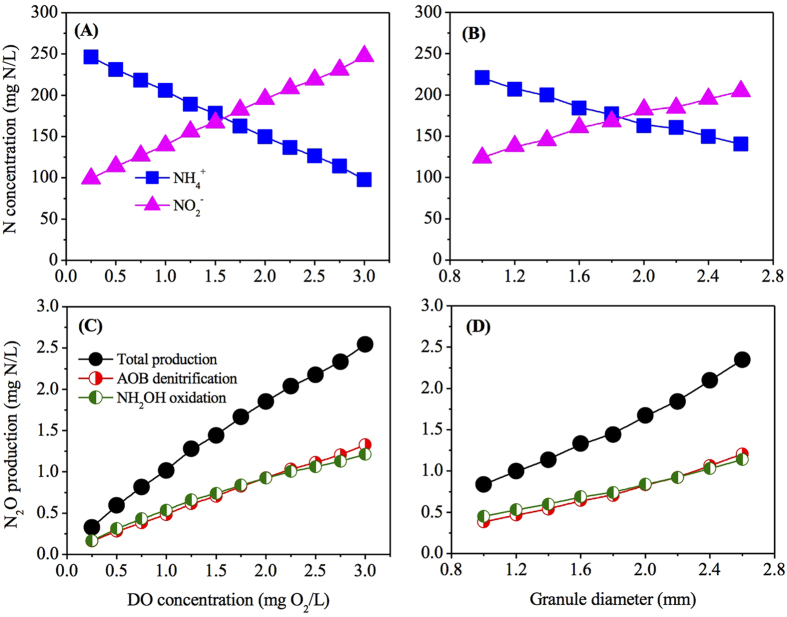
The effect of DO (**A**,**C**) and granule size (**B**,**D**) on reactor performance and N_2_O production based on the two-pathway N_2_O model.

Model simulations for the PN granules with bulk oxygen ranging from 0.25 to 3.0 mg O_2_/L and diameters in the range from 1.0 to 2.6 mm were conducted to evaluate the effects of DO and granule size on N_2_O production from the granular PN system. Figure 3A,C show the simulation results of effluent N and N_2_O production at different bulk oxygen concentrations. With the increase of bulk oxygen concentration from 0.25 to 3.0 mg O_2_/L, the effluent NH_4_^+^ concentration decreased from ~250 to ~100 mg N/L, accompanied by corresponding increase of effluent NO_2_^−^ (Fig. 3A). Higher N_2_O production was observed in PN-SBR with higher bulk oxygen levels (Fig. 3C), provided that both AOB denitrification and NH_2_OH oxidation pathways were stimulated by the increased DO concentration. Higher oxygen concentration leads to higher AOR, resulting in a higher electron flow, which would increase N_2_O production from both incomplete NH_2_OH oxidation and nitrite reduction. At DO level of 2.0 mg O_2_/L, the contributions of the two pathways were identical. At higher DO levels, AOB denitrification pathway contributed a bit more to N_2_O production (~52% at DO of 3.0 mg O_2_/L), whilst NH_2_OH oxidation pathway made more contribution at lower DO levels (~51% at DO of 0.25 mg O_2_/L). The model prediction with regard to the shift of pathway under varying DO conditions was inconsistent with the observations in previous studies^3^,^13^,^34^. It is known that higher DO stimulates N_2_O production via affecting ammonia oxidation rate (AOR)^35^. The affinity constant of Mred for NO reduction (*K*_*mred*,2_ = 1 × 10^−5^ mmol/g-VSS) is much lower than that for nitrite reduction (*K*_*mred*,4_ = 1.9 × 10^−1^ mmol/g-VSS), indicating a higher competition for electron of the NO reduction process. However, higher bulk DO would also result in higher nitrite accumulation, which in turn exerts an inhibitory effect on N_2_O production^36^. It is known that nitrite affects the two N_2_O production pathways differently^11^,^26^. And high nitrite concentration could exert an inhibitory effect on N_2_O production in a PN bioreactor^36^, which was consistent with the observations in our study. Furthermore, the inhibition constant for NO reduction (*K*_*I*,2_ = 60 mg N/L) is one magnitude lower than that for nitrite reduction (*K*_*I*,1_ = 500 mg N/L), suggesting that the inhibition of nitrite accumulation on NH_2_OH oxidation pathway is more obvious than the inhibition on AOB denitrification pathway. This was also confirmed by the model simulation in Fig. 1D, where the contribution of NH_2_OH oxidation decreased as nitrite concentration increased. The relative contribution of the two known pathways to N_2_O production predicted by the granule-based N_2_O model in Fig. 3C was a consequence of the combined effect of DO and nitrite.

The simulation results of effluent N and N_2_O production at varying granule diameters are presented in Fig. 3B,D. With the increase of granule diameter from 1.0 to 2.6 mm, the effluent NH_4_^+^ concentration decreased from ~220 to ~140 mg N/L, accompanied by corresponding increase of effluent NO_2_^−^ (Fig. 3B). The N_2_O production via two pathways is almost linearly dependent on the granule diameter (Fig. 3D). With the granule number keeping constant, the increase of granule diameter increased the overall granule/liquid interfacial area, thus induced higher reaction rate^37^. However, it should be noted that no biomass growth or decay were considered in this granule-based model for simplification. In reality, the substrate limitation caused by diffusion resistance in the granule would lead to cell decay and leakage of inert cellular products^38^. Consequently, larger granules with higher diffusion resistance contained a higher inert fraction and thereby a decreased volumetric activity^38^. The increase of granule size would result in higher reaction rate, but lower volumetric activity. Stable effluent N would be achieved once they were in equilibrium. In granule diameter range of 1–2.2 mm, NH_2_OH oxidation pathway made more contribution under the simulated conditions (50–54%), while AOB denitrification pathway contributed a bit more to overall N_2_O production in the range of 2.2–2.6 mm (50–51%) (Fig. 3D).

**Figure 4:**
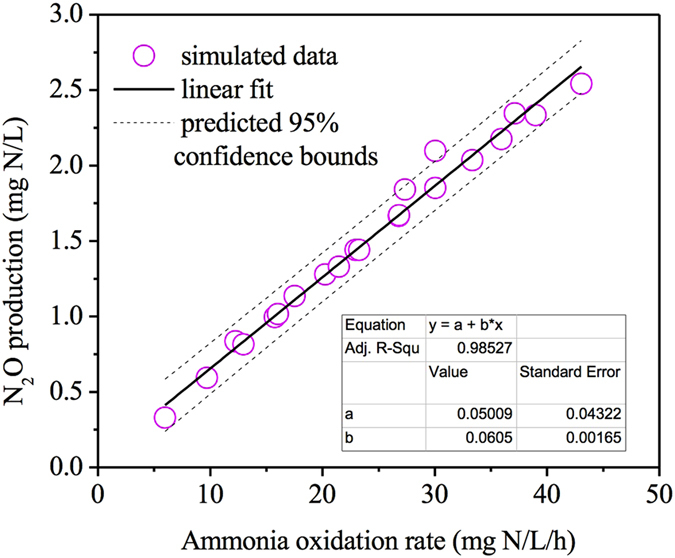
The correlation between N_2_O production and ammonia oxidation rate under the conditions of different DO levels and granule sizes based on simulation results.

Figure 4 summarizes the N_2_O production at each of the corresponding AOR under various conditions with different DO and granule diameters applied. The N_2_O production increased almost linearly from 0.33 to 2.54 mg N/L as the AOR increased from 6.0 to 43.1 mg N/L/h. By conducting linear regression based on the simulation results, it can be seen that most of the data points stayed within the predicted 95% confidence bounds, indicating good linear relationship between N_2_O concentration and AOR within the tested range. The correlation between N_2_O and AOR identified in this study was consistent with previous studies using PN-SBR systems^9^,^21^. Law *et al*.^9^ observed a linear correlation between N_2_O and AOR within the pH range of 6.0–8.5. pH likely induced a change in the AOR, which may have in turn affected the N_2_O production rate. Rathnayake *et al*.^21^ positively correlated N_2_O production rate obtained from batch experiments to the corresponding AOR (R^2^ = 0.71). Electrons are made available at a higher rate under a higher AOR and diverted to the both nitrite reduction and NO reduction for N_2_O production, since AOB do not possess the capability for N_2_O reduction to N_2_^39^. However, some studies revealed an exponential relationship between N_2_O and AOR, with DO and NH_4_^+ ^35 or IC^12^ as the factors to vary AOR. The experimental results were further used to develop a mathematical model considering a non-enzymatic N_2_O production pathway by AOB^35^ and an integrated two-pathway model with consideration of catabolic and anabolic processes^25^, respectively. Based on the model predictions, it was seen that higher AOR would cause accumulation of intracellular intermediates such as the unstable nitrosyl radical (NOH), which lad to their faster breakdown to form N_2_O. The relationship between N_2_O and AOR relies largely on the involved N_2_O production pathways. The biological pathways (nitrite reduction or NO reduction) result in linear relationship, whereas the chemical pathway (chemical decomposition of NOH) leads to exponential correlation. However, it is yet to be fully clarified if NO or NOH are the direct source of N_2_O in NH_2_OH pathway, which require further investigation. This is the first study revealing the linear correlation between N_2_O production and AOR in granular bioreactors with varying DO levels and granule sizes. The model simulations are yet to be verified by experimental data obtained from nitritation granular sludge process.

In summary, a two-pathway N_2_O model incorporating both AOB denitrification and NH_2_OH oxidation pathways was applied to describe a granule-based PN system and provided insights into the N_2_O production mechanisms involved. The main conclusions are listed below:

- The two-pathway N_2_O model could well capture the experimentally measured N_2_O dynamics and pathway distributions in the granule-based PN-SBR.
- The substrate diffusion (i.e. NH_2_OH, oxygen and electron carriers) throughout the granule determined the N_2_O distribution inside the granule. Both pathways made comparable contribution at the granule surface, while AOB denitrfication pathway was dominant over NH_2_OH oxidation pathway inside the granule.
- N_2_O production via both pathways was positively correlated to both the bulk oxygen and the granule diameter. N_2_O production was linearly dependent on AOR under the conditions of varying bulk oxygen levels and granule diameters.

## Materials and Methods

### Two-pathway N_2_O model

The two-pathway model for prediction of N_2_O production by AOB was previously established by Ni *et al*.^26^. The N_2_O model integrates both AOB denitrification and NH_2_OH oxidation pathways and synthesizes relevant biochemical reactions in conversion of ammonia (NH_3_), NH_2_OH, NO_2_^−^, NO, N_2_O and O_2_. The key feature of the two-pathway N_2_O model of AOB is that the model decouples the oxidation and reduction processes through a pool of electron carriers. The electron transfer from oxidation to reduction is modeled by introducing electron mediators (Mred and Mox) as the new state variables. The definition and unit for these substrates are presented in Table S1 in Supplementary Material.

Table S2 summarized the kinetics and stoichiometry of the model. The electrons, generated by incomplete oxidation of NH_2_OH to NO_2_^−^ via NO (Process 2&3) are donated to the oxidation of NH_3_ to NH_2_OH (Process 1), O_2_ reduction to H_2_O (Process 5) and the two N_2_O production pathways, including NO reduction to N_2_O (NH_2_OH oxidation pathway, Process 4) and nitrite reduction to N_2_O (AOB denitrification pathway, Process 6). It should be noted that the nitrite reduction (Process 6) is described as a one-step process without NO intermediate to avoid NO and NO_2_^−^ loop, which is justifiable due to the fact that NO accumulation/emission is rarely observed during denitrification by AOB or heterotrophic denitrifiers^40^. All of the processes above are accompanied by the conversion of Mred to Mox or vice versa. In process 7, an increase of Mred is balanced by a decrease of Mox and vice versa (Mred ⇋ Mox + 2e^−^ + 2 H^+^), with the total level of electron carriers (C_tot_) being constant.

### The granule-based reactor model

The general assumptions made in the granule-based reactor model of this work include: (1) the granules are spherical in shape and uniform in size; (2) the number and size of granules are constant in time; (3) no biomass growth, attachment or detachment and a uniform distribution of AOB throughout the granules for all cases. (4) only radial diffusion transport is considered and is described by Fick’s law; and (5) the diffusion coefficient is constant.

Both oxygen and ammonium are supplied into the granules from the bulk liquid. The aerobic granular reactor is modeled through consisting of a completely mixed gas compartment and a biofilm compartment (containing granules and bulk liquid). The gas compartment is linked to the bulk liquid in the biofilm compartment through diffusive links. The oxygen concentration in the gas compartment is dependent on the gas flow rate and the applied gas pressure. The following kinetics (Eq. 1) is used to model the flux of oxygen (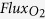) from the gas to the biofilm matrix compartment:


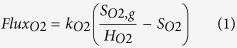


where 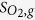 and 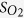 are the concentrations of oxygen in the gas and biofilm matrix compartments (g m^−3^), respectively, 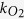 is the overall mass transfer coefficient of oxygen (0.312 m d^−1^)^41^ and 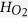 is the Henry coefficient for oxygen (mole O_2_ m^−3^ gas/mole O_2_ m^−3^ liquid).

Parameters with regard to the mass transfer coefficients for the substrates (e.g. ammonium, nitrite, oxygen etc.) are adopted from Hao *et al*.^42^. For the soluble components involved in the biological reactions, the first step is their diffusion into the granules where the reactions take place. Discretization in time of the partial-differential equation describing the reaction-diffusion kinetics in a spherical particle (i.e., granule) is described as the following equation:


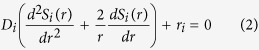


with two boundary conditions:


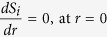



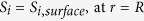


where *S*_*i*_ is the substrate concentration of component i in the granule, *S*_*i,surface*_ is the substrate concentration of component i in the granule surface, *r*_*i*_ is the volumetric substrate conversion rate in the granule, *D*_*i*_ is the diffusion coefficient of substrate i, *r* is the distance from the granule center and R is the granule radius.

It should be noted that no stripping process is considered in the model framework for simplicity as the main objective is to provide insights into involved N_2_O production mechanism in granular sludge process rather than to predict the gas phase emissions. Consequently, we use N_2_O concentration (mg N/L) in bulk liquid to represent the total N_2_O production by the AOB granules under varying operational conditions.

### Experimental data for model testing

Experimental data for model evaluation were obtained from a lab-scale autotrophic PN-SBR previously reported in Rathnayake *et al*.^17^. The lab-scale PN-SBR had working volumes of 2 L and was operated with a cycle time of 4 hours, consisting of 3 min feeding, 232 min aeration, 3 min settling and 2 min decanting. For each cycle, 1 L synthetic wastewater containing 210 mg N/L NH_4_^+^ and 140 mg N/L NO_2_^−^ without organic carbon was fed into the SBR, resulting in a hydraulic retention time (HRT) of 8 hours. The incubation temperature was maintained at 35 °C. The influent pH was around 7.7. Airflow rate was fixed at 0.2 L/min at steady state. The nitrogen loading rate was averaged 43 ± 2.7 mg N/L/h. The granules grown in this PN-SBR had an average diameter of 2 mm. After reaching steady state, the N_2_O concentrations in the off-gas from the reactor were determined once every minute during one typical 4-h cycle with a 1412 Photo acoustic Field Gas-Monitor (INNOVA, Copenhagen, Denmark). The dissolved N_2_O (D-N_2_O) concentration in bulk liquid of the reactor was also measured during one typical 4-h cycle with a N_2_O microsensor (Unisense, Aarhus, Denmark). Meanwhile, samples were taken during the same cycle and measured on an isotope-ratio monitoring mass spectrometer (MAT 252; Thermo Fisher Scientific K.K, Yoko-hama, Japan) for analysis of isotopomer ratios in N_2_O in the off-gas from the SBR. ^15^N-site preference as an illustrative parameter of inter-molecular distribution of ^15^N was calculated as the difference between alpha and beta isotopomer signatures. Characteristic SP values of 0 per mil and 33 per mil for AOB denitrification pathway and NH_2_OH oxidation pathway, respectively, which were estimated in specific pure cultures, were used for estimation of the contribution to each process^43^. Approximate contributions of AOB denitrification pathway and NH_2_OH oxidation pathway to N_2_O production were estimated by assuming that each process is linearly proportional to the SP value.

### Model-based evaluation

The two-pathway N_2_O model by AOB has been previously evaluated using on-line data from both lab-scale and full-scale bioreactors under different operational conditions^11^,^13^,^40^. Unfortunately, a comprehensive dataset including NH_2_OH and electron flux directly obtained from granular reactors is not available. Thus, most of the parameter values were adapted directly from literature, as shown in Table S3, which has been validated in different microbial systems. The selection of these parameters for calibration was on the basis of a sensitivity analysis of the parameters in terms of the measured data. In order for accurate predictions of both nitrogen conversion and N_2_O production in the granule-based PN reactor, a two-step procedure was applied to calibrate the model with the remaining six key sensitive parameters. Firstly, the ammonium oxidation kinetics (i.e.,*r*_*NH*3,*ox*_ and *K*_*O*2,*NH*3_) were calibrated using the ammonium, nitrite and DO data. In second phase, two key parameters for N_2_O production processes by AOB (*r*_*NO*2,*red*_ and *r*_*NO,red*_) were further calibrated using the N_2_O data in liquid and off gas as well as the results based on SP analysis. The experimental data for model testing displayed a decreasing N_2_O production upon nitrite build-up. The inhibitory effect of nitrite on N_2_O production in a PN SBR was also reported by Law *et al*.^36^. Thus, inhibition kinetics (refers to Table S3) were applied to describe both NO reduction and nitrite reduction, in which *K*_*I*,1_ (nitrite inhibition constant for nitrite reduction) and *K*_*I*,2_ (nitrite inhibition constant for NO reduction) were also calibrated. Kinetic control of all the enzymatic reaction rates is described by the Michaelis−Menten equation. The rate of each reaction is modeled by an explicit function of the concentrations of all substrates involved in the reaction. The N_2_O production rates from the two pathways are shown in the following equations, respectively:


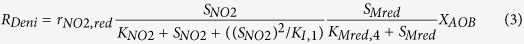


where *R*_*Deni*_ represents N_2_O production rate via AOB denitrification pathway; *r*_*NO*2,*red*_ is specific maximum nitrite reduction rate; *K*_*NO*2_ is nitrite affinity constant for nitrite reduction; *K*_*Mred*,4_ is *S*_*Mred*_ affinity constant for nitrite reduction; *K*_*I*,1_ is nitrite inhibition constant for nitrite reduction; *S*_*NO*2_ and *S*_*Mred*_ are the soluble nitrite concentrations; *X*_*AOB*_ is active biomass concentration of AOB.


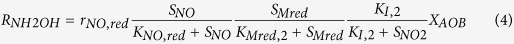


where *R*_*NH*2*OH*_ represents N_2_O production rate via NH_2_OH oxidation pathway; *r*_*NO,red*_ is specific maximum NO reduction rate; *K*_*NO,red*_ is NO affinity constant for NO reduction; *K*_*Mred*,2_ is *S*_*Mred*_ affinity constant for NO reduction; *K*_*I*,2_ is nitrite inhibition constant for NO reduction; *S*_*NO*_ is the soluble NO concentration.

The initial values, adopted from literature, together with restriction ranges of these six parameters were shown in Table S4. The parameter values were estimated by minimizing the sum of squares of the deviations between the measured data and the model predictions using the secant method embedded in AQUASIM^44^. The secant optimization method is well suited for the minimization of numerically integrated equations using linear approximation of the model functions, which can lead to a much faster end convergence being close to a well-defined minimum. Parameter uncertainty evaluation was done according to Batstone *et al*.^45^. The standard errors and 95% confidence intervals of individual parameter estimates were calculated from the mean square fitting errors and the sensitivity of the model to the parameters. The determined F-values were used for parameter combinations and degrees of freedom in all cases. A modified version of AQUASIM 2.1d was used to determine the parameter surfaces^46^. DO concentration plays a very important role in the PN system performance as well as N_2_O production^35^. The granule diameters also have a significant effect on the simulation results by affecting the overall granule/liquid interfacial area^37^. Hence, Model simulations were then performed under varying conditions (DO in bulk liquid from 0.25–3.0 mg O_2_/L and granule diameters from 1–2.6 mm and) to provide insights into the effects of DO and granule size on N_2_O production from the PN-SBR reactor (Refer to Table S5).

## Additional Information

**How to cite this article:** Peng, L. *et al*. Nitrous Oxide Production in a Granule-based Partial Nitritation Reactor: A Model-based Evaluation. *Sci. Rep.* **7**, 45609; doi: 10.1038/srep45609 (2017).

**Publisher's note:** Springer Nature remains neutral with regard to jurisdictional claims in published maps and institutional affiliations.

## Supplementary Material

Supplementary Materials
