# Supplementary material for: Nitrous Oxide Production in a Granule-based Partial Nitritation Reactor: A Model-based Evaluation
Source: Sci Rep. 2017 Apr 3;7:45609. doi: 10.1038/srep45609 (PMC5377315; doi:10.1038/srep45609)
Supplement: Supplementary Materials [file srep45609-s1.doc]

**Supplementary Material**

**Nitrous Oxide Production in a Granule-based Partial Nitritation Reactor: A Model-based Evaluation**

Lai Peng^b^, Jing Sun^a^, Yiwen Liu^c^, Xiaohu Dai^a^, Bing-Jie Ni^a^,*

^a^State Key Laboratory of Pollution Control and Resources Reuse, College of Environmental Science and Engineering, Tongji University, Shanghai 200092, PR China

^b^Research group of Sustainable Energy, Air and Water Technology, Department of Bioscience Engineering, University of Antwerp, Antwerp 2020, Belgium

^c^Centre for Technology in Water and Wastewater, School of Civil and Environmental Engineering, University of Technology Sydney, Sydney, NSW 2007, Australia

***Corresponding author:**

Bing-Jie Ni, P: +86 21 65986849; F: +86 21 65983602; E-mail: bjni@tongji.edu.cn

**The following is included as supporting information for this paper:**

number of pages: 8

number of tables: 5

number of figures: 1

Table S1. The definition and units of model components

| **Variable** | **Description** | **Unit** |
| --- | --- | --- |
| $S_{NH3}$ | Ammonia concentration | mg N/L |
| $S_{NH2OH}$ | Hydroxylamine concentration | mg N/L |
| $S_{NO_{2}^{-}}$ | Nitrite concentration | mg N/L |
| $S_{NO}$ | Nitric oxide concentration | mg N/L |
| $S_{N2O}$ | Nitrous oxide concentration | mg N/L |
| $S_{O2}$ | Dissolved oxygen concentration | mg O_2_/L |
| $S_{Mred}$ | Mediator concentration (Reduced form) | mmol/g VSS |
| $S_{Mox}$ | Mediator concentration (Oxidized form) | mmol/g VSS |
| $X_{AOB}$ | Active AOB biomass concentration | mg COD/L |

**Table S2.** **Process matrix related to N_2_O dynamics in nitrifying biofilm**

| Process | $S_{O2}$ | $S_{NH3}$ | $S_{NH2OH}$ | $S_{NO_{2}^{-}}$ | $S_{NO}$ | $S_{N2O}$ | $S_{Mox}$ | $S_{Mred}$ | Kinetic rate expressions |
| --- | --- | --- | --- | --- | --- | --- | --- | --- | --- |
| 1. Ammonia oxidation | -1 | -1 | 1 |  |  |  | 1 | -1 | $r_{NH3,ox}\frac{S_{O2}}{K_{O2,NH3}+S_{O2}}\frac{S_{NH3}}{K_{NH3}+S_{NH3}}\frac{S_{Mred}}{K_{Mred,1}+S_{Mred}}X_{AOB}$ |
| 2. NH_2_OH oxidation |  |  | -1 |  | 1 |  | -3/2 | 3/2 | $r_{NH2OH,ox}\frac{S_{NH2OH}}{K_{NH2OH}+S_{NH2OH}}\frac{S_{Mox}}{K_{Mox}+S_{Mox}}X_{AOB}$ |
| 3. NO oxidation |  |  |  | 1 | -1 |  | -1/2 | 1/2 | $r_{NO,ox}\frac{S_{NO}}{K_{NO,ox}+S_{NO}}\frac{S_{Mox}}{K_{Mox}+S_{Mox}}X_{AOB}$ |
| 4. NO reduction |  |  |  |  | -1 | 1 | 1/2 | -1/2 | $r_{NO,red}\frac{S_{NO}}{K_{NO,red}+S_{NO}}\frac{S_{Mred}}{K_{Mred,2}+S_{Mred}}\frac{K_{I,2}}{K_{I,2}+S_{NO_{2}^{-}}}X_{AOB}$ |
| 5. Oxygen reduction | -1/2 |  |  |  |  |  | 1 | -1 | $r_{O2,red}\frac{S_{O2}}{K_{O2,red}+S_{O2}}\frac{S_{Mred}}{K_{Mred,3}+S_{Mred}}X_{AOB}$ |
| 6. Nitrite reduction |  |  |  | -1 |  | 1 | 1 | -1 | $r_{NO_{2}^{-},red}\frac{S_{NO_{2}^{-}}}{K_{NO_{2}^{-}}+S_{NO_{2}^{-}}+((S_{NO_{2}^{-}})^{2}/K_{I,1})}\frac{S_{Mred}}{K_{Mred,4}+S_{Mred}}X_{AOB}$ |
| 7. Electron carriers |  |  |  |  |  |  |  |  | $S_{Mred}+S_{Mox}=C_{tot}$ |

**Table S3. Kinetic and stoichiometric parameters of the N_2_O model**

| Parameter | Definition | Values | Unit | Source |
| --- | --- | --- | --- | --- |
| $r_{NH3,ox}$ | Specific maximum ammonia oxidation rate | 0.1 | mg-N/(mg-COD*h) | estimated |
| $r_{NH2OH,ox}$ | Specific maximum NH_2_OH oxidation rate | 0.23 | mg-N/(mg-COD*h) | (2) |
| $r_{NO,ox}$ | Specific maximum NO oxidation rate | 0.23 | mg-N/(mg-COD*h) | (2) |
| $r_{O2,red}$ | Specific maximum oxygen reduction rate | 1.42 | mg-O_2_/(mg-COD*h) | (1) |
| $r_{NO_{2}^{-},red}$ | Specific maximum nitrite reduction rate | 0.077 | mg-N/(mg-COD*h) | estimated |
| $r_{NO,red}$ | Specific maximum NO reduction rate | 0.0059 | mg-N/(mg-COD*h) | estimated |
| $K_{O2,NH3}$ | O_2_ affinity constant for ammonia oxidation | 5 | mg-O_2_/L | estimated |
| $K_{NH3}$ | NH_3_ affinity constant for ammonia oxidation | 2.4 | mg-N/L | (2) |
| $K_{NH2OH}$ | NH_2_OH affinity constant for its oxidation | 0.7 | mg-N/L | (2) |
| $K_{NO,ox}$ | NO affinity constant for NO oxidation | 0.0084 | mg-N/L | (2) |
| $K_{O2,red}$ | O_2_ affinity constant for oxygen reduction | 0.06 | mg-O_2_/L | (2) |
| $K_{NO_{2}^{-}}$ | Nitrite affinity constant for nitrite reduction | 0.14 | mg-N/L | (2) |
| $K_{NO,red}$ | NO affinity constant for NO reduction | 0.0084 | mg-N/L | (2) |
| $K_{Mox}$ | *S_Mox_* affinity constant for NO oxidation | 0.01 | mmol/g-VSS | (2) |
| $K_{Mred,1}$ | *S_Mred_* affinity constant for ammonia oxidation | 1×10^-3^×C_tot_ | mmol/g-VSS | (2) |
| $K_{Mred,2}$ | *S_Mred_* affinity constant for NO reduction | 1×10^-3^×C_tot_ | mmol/g-VSS | (2) |
| $K_{Mred,3}$ | *S_Mred_* affinity constant for oxygen reduction | 6.9×10^-2^ | mmol/g-VSS | (2) |
| $K_{Mred,4}$ | *S_Mred_* affinity constant for nitrite reduction | 1.9×10^-1^ | mmol/g-VSS | (2) |
| $K_{I,1}$ | Nitrite inhibition constant for nitrite reduction | 500 | mg-N/L | estimated |
| $K_{I,2}$ | Nitrite inhibition constant for NO reduction | 60 | mg-N/L | estimated |
| $C_{tot}$ | The sum of *S_Mred_* and *S_Mox_*, a constant | 1×10^-2^ | mmol/g-VSS | (2) |
| Source: (1) Ni et al., 2015; (2) Ni et al., 2014 | | | | |

**Table S4. Best-f**it values, restriction range and initial values of the six estimated parameters.

| Parameter | Estimated value | Restriction range | Initial value |
| --- | --- | --- | --- |
| $r_{NH3,ox}$ | 0.1 | 0-1 | 0.21 |
| $r_{NO_{2}^{-},red}$ | 0.077 | 0-1 | 0.043 |
| $r_{NO,red}$ | 0.0059 | 0-0.1 | 0.00022 |
| $K_{O2,NH3}$ | 5 | 0-10 | 0.61 |
| $K_{I,1}$ | 500 | 1-1000 | 48.3 |
| $K_{I,2}$ | 60 | 1-100 | 10 |

Table S5. Summary of model simulation scenarios.

| DO Concentration (mg O2/L) | Granule Diameter (mm) | N concentration N_2_O production (mg N/L) |
| --- | --- | --- |
| 0.25, 0.5, 0.75, 1.0, 1.25, 1.5, 1.75, 2.0, 2.25, 2.5, 2.75, 3.0 | 1, 1.2, 1.4, 1.6, 1.8, 2.0, 2.2, 2.4, 2.6 | Simulation results |

**Figure S1.** 95% confidence regions for the parameter combinations among the key model parameters for the N_2_O production processes by AOB with the best fits in the center, as well as their standard errors: A. $K_{O2,NH3}$ vs. $r_{NH3,ox}$; B. $r_{NO_{2}^{-},red}$ vs. $r_{NO,red}$.

**References:**

Ni, B.J., Pan, Y., van den Akker, B., Ye, L., Yuan, Z., 2015. Full-Scale Modeling Explaining Large Spatial Variations of Nitrous Oxide Fluxes in a Step-Feed Plug-Flow Wastewater Treatment Reactor. *Environmental Science & Technology* 49, 9176-9184.

Ni, B.J., Peng, L., Law, Y., Guo, J., Yuan, Z., 2014. Modeling of nitrous oxide production by autotrophic ammonia-oxidizing bacteria with multiple production pathways. *Environmental Science & Technology* 48, 3916-3924.
